# Exploring the multidimensional factors associated with the incidence of bacillary dysentery in China based on a panel data model

**DOI:** 10.3389/fpubh.2026.1701162

**Published:** 2026-03-09

**Authors:** Yanbin Hao, Dinglin Yu

**Affiliations:** 1 Department of Health Statistics, School of Public Health and Health Management, Gannan Medical University, Ganzhou, Jiangxi, China; 2 State Key Laboratory of Resources and Environmental Information System, Institute of Geographic Sciences and Natural Resources Research, Chinese Academy of Sciences, Beijing, China

**Keywords:** bacterial dysentery, panel data model, socioeconomic factors, forest cover, meteorological factors

## Abstract

**Objective:**

Bacillary dysentery (BD) remains a public health concern in China, with its incidence influenced by multidimensional and interconnected factors. This study aimed to quantify the correlations between meteorological conditions, socioeconomic status, health resources, forest coverage, and BD incidence—an integrated analysis lacking in previous research. The results provide a comprehensive evidence base for formulating targeted BD prevention strategies.

**Methods:**

Using panel data from 31 Chinese provinces (2014 ~ 2018), including BD incidence rates and 12 indicators of proxy variables at the ecological level, fixed-effects and random-effects models with Driscoll–Kraay robust standard errors were applied.

**Results:**

The results of the random-effects model incorporating multidimensional factors showed that a 1% increase in the number of beds per 1,000 people in healthcare facilities, GDP per capita, and forest coverage was significantly associated with decreases in BD incidence rates of 2, 0.8, and 0.6%, respectively, (all *p* < 0.05). These findings indicate ecological associations with the incidence of BD across different provinces in China.

**Conclusion:**

Improvements in regional investment in healthcare resources, economic development, and forest coverage were inversely associated with BD incidence, suggesting potential protective effects at the population level.

## 1 Introduction

Bacillary dysentery (BD) is an intestinal infectious disease caused by the *bacterium Shigella dysenteriae* (*Shigella* spp.). This disease is mainly transmitted through the digestive tract, leading to a short duration of post-disease immunity and, consequently, an increased risk of reinfection in the short term. It is one of the most common diarrheal diseases in clinical practice (1–3). According to the data released by the World Health Organization, a total of 175,198 cases of dysentery were reported globally in 2019, with BD accounting for the highest percentage of cases (99.5%). Dysentery is primarily prevalent in developing countries and regions in Asia, Africa, and Latin America. China has been one of the countries with a high incidence of dysentery. However, in recent years, there has been a downward trend in dysentery cases. In 2021, domestic bacterial dysentery ranked ninth among Category A and B notifiable infectious diseases, but it is still higher than the levels reported in developed countries (4).

It has been shown that the incidence of BD is associated with regional meteorological factors, economic development, health resource investment, urbanization, and education levels (5–8). While individual studies have linked BD incidence to meteorological variables such as temperature, humidity, and rainfall, the majority of these studies have focused on single-factor correlations rather than multi-dimensional interactions. Climate change plays a significant role in the occurrence and transmission process of infectious diseases, showing a positive correlation between temperature and the incidence of BD, which will gradually increase with rising temperature, relative humidity, and rainfall. The decline in the incidence of BD is also considered to be related to economic development, higher levels of education and investment in health resources, increased awareness of health and hygiene, and improved sanitation practices (4, 6, 9). Socioeconomic development, which includes factors such as GDP per capita, water resources, and education levels, further modulates population vulnerability by influencing hygiene practices and health awareness; however, these factors are often analyzed in isolation from ecological and health resource contexts. Healthcare infrastructure, such as medical bed supply and health technician density, plays a critical role in early intervention and transmission control, but its synergistic effects on economic and ecological factors remain understudied.

After an extensive literature review, we found that few studies have simultaneously considered the four dimensions of factors—meteorological conditions, socioeconomic status, health resources, and forest coverage—to analyze their impact on BD incidence. Specifically, there were more studies exploring the associations between the first three factors and infectious diseases. In contrast, studies investigating the correlation between forest or vegetation coverage and BD remain scarce (10, 11).

The theoretical framework of the study was based on the intersection of ecology and epidemiology, integrating various factors. It was believed that the transmission of BD results from the interaction of environmental factors (such as temperature, humidity, rainfall, and sunshine), socioeconomic status indicators (such as GDP per capita and population density), environmental characteristics (such as the vegetation coverage index and forest coverage rate), and the distribution of medical resources (such as the number of types of hospitals and the number of beds). Specifically, meteorological factors indirectly affect the occurrence of diseases by affecting the survival and transmission efficiency of pathogens. Social and economic factors regulate the risk of diseases by affecting health habits, living conditions, and the accessibility of medical services. Environmental characteristics not only provide habitats for pathogens but also modify the risk of exposure by influencing patterns of human activity. The accessibility (12) and quality (13) of medical resources directly determine the efficiency of case detection and treatment, thereby affecting the dynamics of disease transmission.

Generally, analyzing the influence of multiple factors on the epidemics of infectious diseases across different times and regions requires the construction of statistical models. Meta-analysis effectively synthesizes multi-factor evidence in complex systems (14), while robust feature selection enhances the reliability of high-dimensional health data (15). Time-series ensemble models highlight the importance of comprehensive frameworks in understanding infectious diseases (16), and advanced inference tools offer insights for optimizing variable analysis (17). Despite individual BD factor verification, few studies adopt integrated multivariable and robust modeling approaches developed in the health and environmental domains. This gap limits targeted prevention, particularly in regions with heterogeneous conditions.

Furthermore, the majority of existing studies relied solely on time-series analysis models (ignoring regional differences) or cross-sectional geospatial regression models (ignoring temporal trends) (18, 19); however, the characteristics of heterogeneity or the degree of autocorrelation may vary across different time scales. This makes it challenging to comprehensively consider spatiotemporal characteristics, leading to relatively independent analyses of factors across different spatial contexts (19). This study used a panel data model that integrates both cross-sectional (spatial) and time-series (temporal) dimensions of the data. A core advantage of the panel data model is its ability to simultaneously control for variables that vary across regions but remain stable over time (e.g., inherent characteristics of each province) and variables that change over time but remain consistent across all regions (e.g., national policy changes and application of general technologies). Meanwhile, the panel data model has unique advantages in controlling for unobserved heterogeneity (20). By introducing individual or time fixed effects, the panel data model can effectively reduce omitted variable bias and improve the accuracy of estimating the effects of key influencing factors on the regional epidemic situation of BD.

In this study, data from 2014 to 2018 were used, including the annual incidence rates of BD in 31 provincial-level administrative regions in China (excluding Hong Kong, Macao, and Taiwan). Additionally, the study incorparated data on local meteorological factors, regional economic development levels, health resource inputs, and educational attainment. A panel data model was used to quantitatively examine the factors associated with the incidence rate of BD, providing a theoretical basis and recommendations for the implementation of population-level health planning.

## 2 Materials and methods

### 2.1 Data on disease and factors

In this study, data on the reported incidence of BD at the provincial level from 2014 to 2018 were obtained from the Public Health Science Data Center (https://www.phsciencedata.cn/Share/index.jsp). This database compiles all confirmed dysentery cases reported through the infectious disease network reporting system from different provinces across the country. BD is a Category B infectious disease in China. Cases are diagnosed based on the unified national diagnostic criteria (WS287-2008). A confirmed case is defined as one that meets the clinical diagnostic criteria and supported by etiological evidence, specifically the isolation of *Shigella* from stool samples. All clinical and hospital physicians are required to report both clinical diagnoses and confirmed BD cases to the local CDC within 24 h through the Internet-based Chinese Disease Prevention and Control Information System.

The average temperature, average relative humidity, total annual precipitation, and total annual sunshine hours were obtained from the China Meteorological Data Network (http://data.cma.cn/). Data on the number of health technicians per 1,000 people, the number of beds per 1,000 people in healthcare institutions, the proportion of urban population, population density, GDP per capita, the proportion of people in higher education, per capita water resources, and forest coverage were obtained from the China Statistical Yearbook, China Health Statistics Yearbook, and China Environmental Statistics Yearbook published from 2015 to 2019. The statistical yearbook released in a given year actually contains data from the previous year.

### 2.2 Methods

#### 2.2.1 Model introduction

The general form of the panel data regression model is: $y_{\mathrm{it}}=\sum_{k=1}^{k}\beta_{\mathrm{ki}}x_{\mathrm{kit}}+u_{it}$ . In the formula, i =1, 2, …, 31, indicates 31 provincial administrations; *t* = 1, 2, …, 5, denotes 5 years, from 2014 to 2018; $y_{\mathrm{it}}$ is the dependent variable, representing the value for the i th region in the *t*th year; $\beta_{\mathrm{ki}}$ is the parameter to be estimated; $x_{\mathrm{kit}}$ is the explanatory variable, indicating the initial value of the *k*th explanatory variable for the i th region in the *t*th year; and $u_{it}$ is the random error term. The fixed-effects model stratifies the intercept term as α + $\alpha_{i}$ , meaning that the intercept terms for different individuals are different. The model form is $y_{\mathrm{it}}=\alpha +\alpha_{i}+\sum_{k=1}^{k}\beta_{\mathrm{ki}}x_{\mathrm{kit}}+u_{it}$ . Here, $\alpha_{i}$ indicates that different individuals have different intercept terms, reflecting each individual’s deviation from the overall mean. The model parameters are primarily estimated by introducing dummy variables, using least squares dummy variable regression to estimate $\alpha_{1},\alpha_{2}$ ,…, $\alpha_{N}$ , one by one. In addition, the random-effects model (REM) divides the intercept term into $\alpha +\upsilon_{i}\;$ and treats the intercept term as a random variable with mean α . The model form is as follows: $\;y_{\mathrm{it}}=\alpha +\sum_{k=1}^{k}\beta_{\mathrm{ki}}x_{\mathrm{kit}}+\upsilon_{i}+u_{it}$ , where $\upsilon_{i}$ reflects the random influence of individual members (21–23).

#### 2.2.2 Variable definition and processing

##### 2.2.2.1 Dependent variable

*Y* represents the annual BD incidence rate in each province (1/100,000 people). In this study, the annual incidence rate was calculated as follows: Annual incidence rate = (the number of new cases in the region in that year/the total population in the region in the same year) × 100%.

##### 2.2.2.2 Independent variables

Based on the epidemiological transmission mechanism of BD and the social–environment–health causal theory, multiple indicators serve as proxy variables at the ecological level. Although these indicators cannot directly measure individual exposure, they are widely used in ecological studies to reflect key characteristics at the regional level.

Meteorological variables (temperature, humidity, precipitation, and sunshine) serve as proxy variables for overall regional environmental exposure levels. The number of health technicians and the number of beds serve as proxy variables for regional health system capacity. GDP per capita, population density, and the proportion of the urban population serve as proxy variables for regional socioeconomic development. Forest coverage rate and per capita water resources serve as proxy variables for ecological sanitation and water accessibility. Higher education levels serve as a proxy variable for health literacy and health-related behavior.

The precise definitions and calculations of each variable are as follows:

*X_1_*: Annual average temperature (°C) (7, 8), calculated as the average of all daily temperatures in a year.

*X_2_*: Annual average relative humidity (%) (18).

*X_3_*: Total annual precipitation (mm) (18).

*X_4_*: Total annual sunshine hours (hours) (18), calculated as the sum of all hours of sunshine per year in a certain area.

*X_5_*: Number of health technicians per 1,000 people (9), calculated as Number of health technicians per 1,000 people = (number of health technicians/total population) × 1,000.

*X_6_*: Number of beds in medical and health institutions per 1,000 people (9), calculated as Number of beds in medical and health institutions per 1,000 people = (number of beds in medical and health institutions/number of total population) × 1,000.

*X_7_*: Proportion of the urban population (%) (8), calculated as Urban population proportion = (urban population/total population) × 100%.

*X8*: Population density (people/km^2^) (18), calculated as Population density = total population/land area.

*X_9_*: GDP per capita (10,000 yuan) (9), calculated as GDP per capita = total GDP/total population.

*X_10_*: Proportion of the population with higher education (%) (9), calculated as Higher education population ratio = (number of higher education students in school/total population) × 100%.

*X_11_*: Per capita water resources (m^3^/person) (9), calculated as Per capita water resources = regional annual total water resources/regional total population.

*X_12_*: Forest coverage rate (%) (24), calculated as Forest coverage rate = (total forest area/total area) × 100%.

The units of the collected raw data differed in magnitude across variables, which could lead to heteroscedasticity (23). To address this issue, all variables were log-transformed and scaled. The transformed variables are denoted as *lnY*, *lnX_1_*, *lnX_2_*, *lnX_3_*, *lnX_4_*, *lnX_5_*, *…*, *lnX_11_*, *lnX_12_*. Assuming that all independent variables are included in the regression model, a multivariate double-log regression model was constructed as follows: $\begin{array}{l} \ln Y_{\mathrm{it}}=\beta_{0}+\beta_{1}\ln X_{1\mathrm{it}}+\beta_{2}\ln X_{2\mathrm{it}}+\beta_{3}\ln X_{3\mathrm{it}}+\beta_{4}\ln X_{4\mathrm{it}}\\ +\beta_{5}\ln X_{5\mathrm{it}}+\beta_{6}\ln X_{6\mathrm{it}}+\ldots +\beta_{11}\ln X_{11\mathrm{it}}+\beta_{12}\ln X_{12\mathrm{it}}, \end{array}$ where $\beta_{i}$ represents the degree of influence of the explanatory variable on the explained variable, that is, the average percentage change in *Y* when *X_i_* changes by 1%.

### 2.3 Statistical analysis

All statistical analyses were performed using Stata 17.0 and R 4.3.2. Descriptive analysis was performed first, followed by the Pearson’s correlation analysis and univariate panel regression analysis to preliminarily assess the correlation between each factor and the incidence of BD. The variance inflation factor (VIF) was used to assess the severity of multicollinearity, with a VIF value greater than 10 indicating a serious multicollinearity problem. In addition, the Pesaran’s CD test, Wooldridge test, and Breusch–Pagan heteroscedasticity test were performed to verify the assumed conditions of the data. The *F* test, BP test, and Hausman test were used to select the optimal model and to perform parameter estimation and interpret the results. The final model was further evaluated through residual diagnostics and assessments of model fit.

We report the results for three broad categories of variables to identify the relatively robust influencing factors. Model 1 included all independent variables, Model 2 excluded socioeconomic factors, and Model 3 excluded meteorological variables.

Based on the data characteristics of this study, robust weighted standard errors were applied, and Driscoll–Kraay robust standard errors (25) were used for subsequent model estimation results. This method provides standard error estimates, which are highly robust to very common spatial–temporal correlations and heteroscedasticity in panel data. Differences were considered statistically significant at a *p*-value of ≤ 0.05.

## 3 Results

### 3.1 Descriptive analysis

#### 3.1.1 Changing trends in the incidence of BD in 31 provinces of China

**Figure 1:**
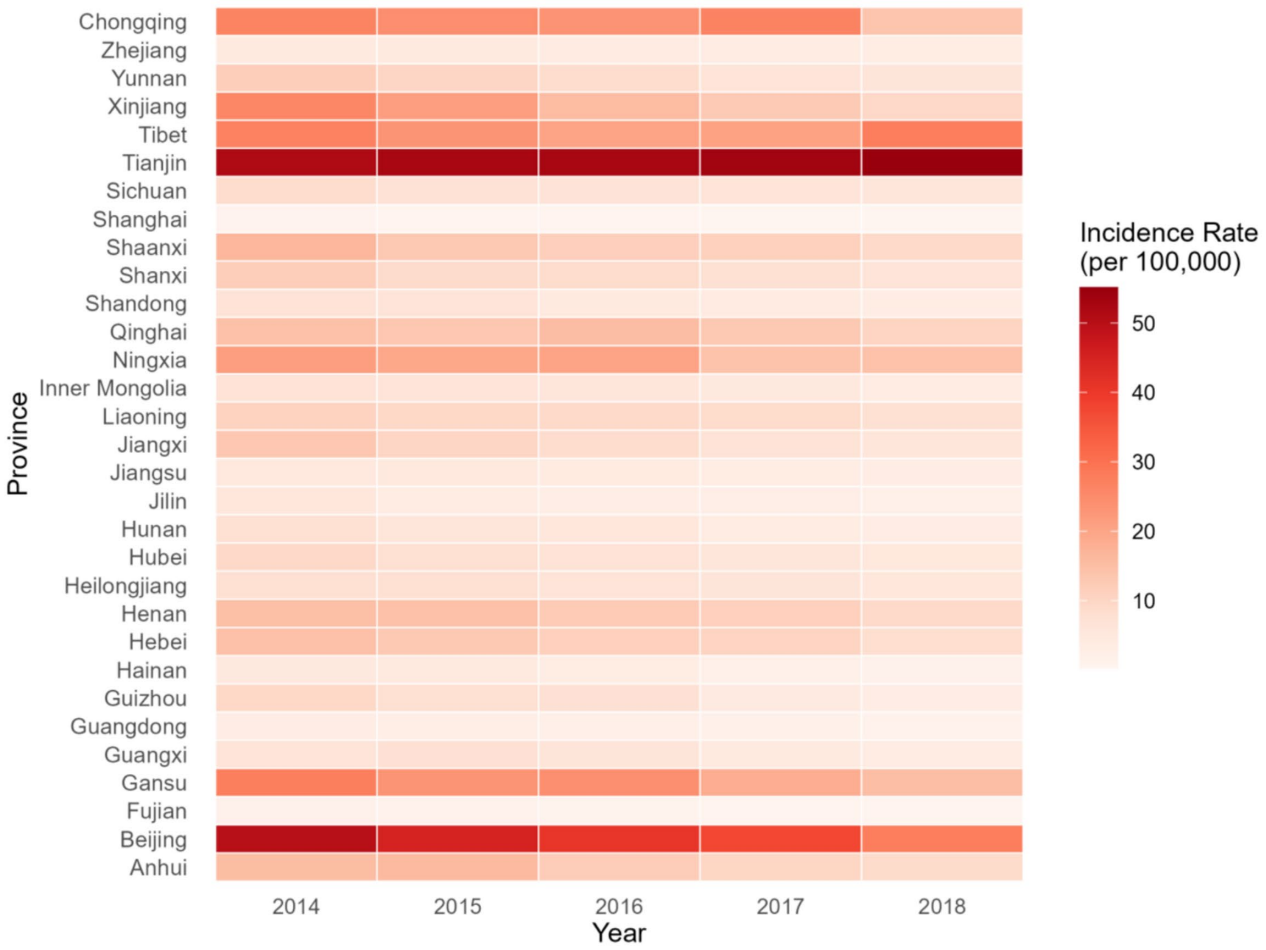
Trend of bacillary dysentery incidence rates across 31 provinces in China from 2014 to 2018.

Figure 1 shows the trend of BD incidence rates across various provinces in China from 2014 to 2018. There was a decreasing trend in nationwide BD incidence over the years, with the exception of Tianjin, which showed a slight upward trend.

#### 3.1.2 Basic information of each variable

Table 1 presents descriptive statistics, including the mean, standard deviation (SD), maximum, minimum, and median for each variable. Some variables, such as the annual incidence rate of BD, total annual precipitation, population density, and per capita water resources, exhibited relatively scattered distributions, and there might be a right-skewed distribution. Data transformation (such as logarithmic transformation) was needed to improve the normality of the data.

**Table 1:** Summary statistics of each variable.

Variable	Notation	Mean	SD	Min.	Max.	Median
Annual incidence rate of bacillary dysentery (1/100,000 people)	*Y*	11.7	11.5	0.3	55.1	8.1
Average temperature (°C)	*X_1_*	14.6	5.1	5.0	25.3	15.6
Average relative humidity (%)	*X_2_*	65.8	12.3	34.3	83.8	64.1
Total annual precipitation (mm)	*X_3_*	988.3	587.8	169.2	2,940	880.2
Total annual sunshine hours (hours)	*X_4_*	2044	560.8	598.4	3,112	2045
Number of health technicians per 1,000 people (person)	*X_5_*	6.3	1.2	4.1	11.9	6.1
Number of beds in medical and health institutions per 1,000 people (beds)	*X_6_*	5.4	0.8	3.8	7.2	5.4
Proportion of the urban population (%)	*X_7_*	57.8	12.5	25.8	89.6	56.0
Population density (person/km^2^)	*X_8_*	460.9	697.0	2.6	3,826	275.5
GDP per capita (10,000 yuan)	*X_9_*	5.7	2.6	2.6	14.1	4.8
Proportion of population with higher education (%)	*X_10_*	14.3	7.5	2.6	48.7	12.8
Per capita water resources (m^3^/person)	*X_11_*	6,478	23,939	76.1	142,311	1,697
Forest coverage rate (%)	*X_12_*	32.8	18.0	4.2	66.8	35.8

### 3.2 Correlation analysis

Correlation analysis was conducted between each pair of variables, as shown in Table 2. It can be seen from the table that the incidence rate of BD was correlated with average temperature, average relative humidity, total annual precipitation, total annual sunshine duration, proportion of urban population, population density, GDP per capita, and forest coverage rate. Except for average temperature, the number of health professionals per 1,000 people, and the number of hospital beds in medical institutions per 1,000 people, the correlation coefficients between the other variables exceeded 0.5. Moreover, the correlation coefficients between population density and GDP per capita and between population density and the proportion of the population with higher education both exceeded 0.8. Therefore, the correlation between the variables was relatively high, which could have a certain impact on the results of the subsequent regression analysis.

**Table 2:** Correlation coefficient matrix.

Notation	Variable	*lnY* (incidence of bacillary dysentery)	*lnX_1_*	*lnX_2_*	*lnX_3_*	*lnX_4_*	*lnX_5_*	*lnX_6_*	*lnX_7_*	*lnX_8_*	*lnX_9_*	*lnX_10_*	*lnX_11_*	*lnX_12_*
*lnX_1_*	Temperature	−0.305^***^	1											
*lnX_2_*	Relative humidity	−0.475^***^	0.625^***^	1										
*lnX_3_*	Precipitation	−0.528^***^	0.752^***^	0.794^***^	1									
*lnX_4_*	Sunshine hours	0.305^***^	−0.624^***^	−0.780^***^	−0.680^***^	1								
*lnX_5_*	Number of health technicians	−0.099	−0.025	−0.115	−0.124	0.150^*^	1							
*lnX_6_*	Number of beds in medical and health institutions	−0.048	−0.336^***^	−0.001	−0.230^***^	−0.020	0.446^***^	1						
*lnX_7_*	Proportion of the urban population	−0.215^***^	0.185^**^	0.200^**^	0.174^**^	−0.067	0.627^***^	0.040	1					
*lnX_8_*	Population density	−0.270^***^	0.629^***^	0.500^***^	0.463^***^	−0.421^***^	0.268^***^	−0.156^*^	0.714^***^	1				
*lnX_9_*	GDP per capita	−0.223^***^	0.213^***^	−0.004	0.185^**^	−0.001	0.662^***^	0.007	0.863^***^	0.567^***^	1			
*lnX_10_*	Proportion of the population with higher education	0.011	−0.091	−0.115	−0.129	0.175^**^	0.747^***^	0.183^**^	0.826^***^	0.445^***^	0.777^***^	1		
*lnX_11_*	Per capita water resources	−0.091	−0.089	0.071	0.254^***^	−0.103	−0.389^***^	−0.021	−0.663^***^	−0.707^***^	−0.476^***^	−0.582^***^	1	
*lnX_12_*	Forest coverage rate	−0.340^***^	0.514^***^	0.559^***^	0.618^***^	−0.508^***^	−0.062	−0.126	0.0970	0.367^***^	−0.0120	−0.175^**^	0.124	1

^*^p < 0.1, ^**^p < 0.05, ^***^p < 0.01.

### 3.3 Test of data conditions

The Pesaran’s CD test yielded a statistic of −0.13 (*p* = 0.8962, *p* > 0.05), indicating no significant cross-sectional correlation. In contrast, the Wooldridge AR (1) test produced an *F* value of 50.517 (*p* = 0, *p* < 0.05), suggesting the presence of first-order autocorrelation. The Breusch–Pagan heteroscedasticity test yielded a BP statistic of 80.335 (*p* = 0, *p* < 0.05), indicating the presence of heteroscedasticity. The optimal lambda value from the Box-Cox analysis was 0.192, which is close to zero and supports the use of logarithmic transformation.

### 3.4 Panel univariate regression analysis

Using the incidence of BD as the dependent variable, single-factor analysis was performed separately for each of the 12 explanatory variables. The results are shown in Table 3. The *p*-values for total sunshine hours, number of health technicians per 1,000 people, number of beds in medical and health institutions per 1,000 people, proportion of the urban population, population density, GDP per capita, proportion of the population with higher education, and forest coverage were all less than 0.05, indicating significant associations with BD incidence. The reason that more factors were significant in the univariate analysis is likely because multivariate analysis had not yet been performed to control for potential confounding variables.

**Table 3:** Univariate panel data analysis of bacillary dysentery incidence across 31 provinces in China.

Variable	*β*	*t*	*p*	*R^2^*
Temperature	−0.674^a^	−1.80	0.075	0.020
Relative humidity	1.107	1.31	0.190	0.016
Precipitation	−0.052	−0.47	0.640	0.001
Sunshine hours	−0.813	−2.23	0.028	0.065
Number of health technicians	−2.664	−8.89	0.000	0.652
Number of beds in medical and health institutions	−2.730	−10.00	0.000	0.693
Proportion of the urban population	−5.417	−7.28	0.000	0.579
Population density	−13.51	−4.94	0.000	0.399
GDP per capita	−1.916	−8.89	0.000	0.635
Proportion of the population with higher education	−0.815	−4.45	0.000	0.212
Per capita water resources	−0.050^a^	−0.58	0.560	0.002
Forest coverage rate	−3.369	−5.84	0.000	0.276

^a^Indicates results based on the random-effects model; all other results are from the fixed-effects model.

### 3.5 Panel multivariate regression analysis

#### 3.5.1 Multicollinearity diagnosis

The 12 indicators were assessed for multicollinearity (Table 4). Multicollinearity may pose a problem when all variables are included simultaneously in Model 1; however, it is not apparent when meteorological variables (Model 2) or socioeconomic variables (Model 3) are considered separately (all VIF values were less than 10).

**Table 4:** Results of the multicollinearity test.

Variable	VIF		
	Model 1	Model 2	Model 3
Temperature	5.37	4.32	—
Relative humidity	8.50	4.36	—
Precipitation	13.06	6.41	—
Sunshine hours	3.13	2.94	—
Number of health technicians	4.60	3.92	3.67
Number of beds in medical and health institutions	2.39	2.17	1.58
Proportion of the urban population	17.37	—	9.42
Population density	23.93	—	5.11
GDP per capita	11.12	—	5.63
Proportion of the population with higher education	6.34	4.16	5.66
Per capita water resources	19.61	2.61	3.94
Forest coverage rate	2.33	1.77	2.19

#### 3.5.2 Panel multivariate regression analysis

We present the regression results of the three incidence models: Model 1, including all independent variables; Model 2, excluding socioeconomic factors; and Model 3, excluding meteorological factors. Among the three regression models in Table 5, only the *p*-values for the number of beds in medical and health institutions per 1,000 people (*X_6_*), GDP per capita (*X_9_*), and forest coverage rate (*X_12_*) were below 0.05, indicating significant ecological associations with the incidence of BD. The Hausman test suggested that the random effects model could be accepted (*p* > 0.05).

**Table 5:** Panel multivariate regression results for the three incidence rate models.

Variable	Fixed effect model (FEM)			Random effect model (REM)		
	Model 1	Model 2	Model 3	Model 1	Model 2	Model 3
Temperature	−0.063	−0.122	—	−0.124	−0.125	—
Relative humidity	−0.203	−0.288	—	−0.264	−0.224	—
Precipitation	−0.044	−0.013	—	−0.043	−0.000	—
Sunshine hours	0.470^*^	0.366	—	0.401^*^	0.370	—
Number of health technicians	0.492	0.045	0.388	0.243	−0.374	0.387
Number of beds in medical and health institutions	−2.332^**^	−2.575^**^	−2.049^*^	−2.205^**^	−2.296^**^	−2.066^**^
Proportion of the urban population	−0.002	—	−0.109	0.258	—	0.233
Population density	1.057	—	0.807	0.156	—	0.001
GDP per capita	−0.808^*^	—	−0.673^*^	−0.888^**^	—	−0.766^*^
Proportion of the population with higher education	−0.022	−0.022	−0.072	−0.008	−0.028	−0.050
Per capita water resources	0.132	0.133	0.041	0.099	0.072	0.005
Forest coverage rate	−1.054^**^	−1.245^**^	−1.060^**^	−0.668^***^	−0.649^**^	−0.693^***^
*R^2^*	0.758	0.737	0.741	0.706	0.667	0.691

^*^p < 0.05, ^**^p < 0.01, ^***^p < 0.001.

Model 1 includes all independent variables; Model 2 excludes socioeconomic factors; Model 3 excludes meteorological variables.

The REM results showed that the regression coefficients for the number of beds in medical and health institutions per 1,000 people, GDP per capita, and forest coverage were approximately −2, −0.8, and −0.6, respectively, across all three models, indicating that these three factors were significantly negatively correlated with the incidence of BD nationwide. While maintaining the other characteristics, a 1% increase in the number of beds in medical and health facilities per 1,000 people, GDP per capita, and forest coverage was associated with a 2, 0.8, and 0.6% decrease in BD incidence rates, respectively.

## 4 Discussion

### 4.1 Model selection

Recently, various methods, such as principal component regression analysis (26), panel data models (27, 28), and GIS-based spatial hierarchical analysis (29), have gradually emerged. Among these, panel data models have been the most popular in the economic and financial fields, as well as in epidemiological research. Panel data consist of observations that have both spatial and temporal dimensions. It helps address the problem of omitted variable bias and often provides large sample sizes and high estimation accuracy.

In the field of public health, early studies commonly used single-factor correlation analysis and multiple regression analysis based on epidemiological data, most of which were cross-sectional or time series analyses. Cross-sectional data analysis examines the distribution and relationships of data at a specific point in time. Compared to panel data models, these methods lack a temporal dimension, ignore individual differences, and neglect the correlation of time series. Time-series data models are suitable for analyzing changes in a single variable over time. However, panel data models are more appropriate for situations involving interactions among multiple factors. They provide more information and stronger analytical capabilities and could reflect changes over time and across individuals. Therefore, panel data models are more suitable for studying certain issues (21–23).

We compared our model with conventional linear regression, and the results showed a significant improvement in model performance (*R^2^* increased from 0.41 to over 0.7). Additionally, we compared the key coefficients of determination with those of other models on other infectious diseases. For example, the *R^2^* of spatial regression models is generally approximately 0.6 (30). In this study, the process of selecting independent variables was guided by a conceptual framework, and measures were taken to minimize multicollinearity.

A previous study explored the spatiotemporal distribution of infectious diseases by incorporating meteorological, socioeconomic, and health resource indicators into a spatial error model. After adjusting for spatial autocorrelation effects, the results indicated that the incidence might be associated with GDP per capita and the number of health technicians but not with meteorological factors (19). These spatial model results are consistent with our findings.

### 4.2 Analysis of related factors

The majority of current studies have shown that the incidence of BD is closely related to meteorological factors, such as temperature, sunshine duration, relative humidity, and total precipitation, which influence the incidence of BD by affecting the survival and transmission of pathogens. For example, it was found that an increase in temperature promotes the reproduction of *Shigella* (31). Medical resource factors, such as the number of health technicians per 1,000 people and the number of beds per 1,000 people, indicate that adequate medical resources can be associated with a reduction in the incidence of BD (24). Social factors, such as the proportion of the urban population and population density, indicate that population density easily increases the risk of transmission (32). Economic and social factors, such as GDP per capita and the proportion of the population with higher education, suggest that improvements in economic and educational levels are conducive to prevention and control (24). Ecological factors, such as per capita water resources and forest coverage rate, suggest that water shortage and low forest coverage may increase the incidence of BD, and these factors may also interact to jointly show correlations with the incidence trend (24, 33, 34).

Regarding the inconsistency between the literature on temperature and humidity and the results of the multivariate model in this study (with no statistical significance or sign changes), we believe that differences in research design may explain this discrepancy. This study used provincial-level panel data and included multiple confounding factors, such as sunshine duration, per capita water resources, and social economy. After controlling for multiple confounding factors, the independent association of temperature and humidity with the incidence of BD was weakened, resulting in no statistical significance or sign changes. In addition, this study covered multiple regions across the country, where regional climate differences were significant and the factors influencing the disease were more complex. The effect of temperature and humidity was diluted by regional heterogeneity, further contributing to the observed differences in the results.

The number of hospital beds per 1,000 people in medical institutions was found to have a negative correlation with the incidence rate of BD. The study results are consistent with those obtained by other researchers (9). The accessibility of medical resources can effectively ensure the early detection and isolation of cases at the population level. This suggests that the number of hospital beds per 1,000 people can be used as an auxiliary indicator for provincial epidemiological monitoring of BD, providing a practical public health reference for optimizing medical resource allocation and strengthening infectious disease prevention and control in key areas.

GDP per capita was negatively correlated with the incidence of BD, which is consistent with the results of other methods (4, 9, 18). Economic development can improve people’s living standards and infrastructure, thereby enhancing the sanitary environment and health literacy. These conditions can effectively reduce the spread of bacterial dysentery. In addition, better medical facilities and higher medical standards enable faster diagnosis and treatment of BD. This conclusion is based on the assumption that the regional economic level is positively correlated with disease prevention and control capacity. It is only applicable for explanations and inferences at the population level. This finding can be used to optimize epidemiological monitoring for bacterial dysentery by using the economic development level as a risk stratification indicator, facilitating precise prevention and targeted interventions.

Forest coverage was negatively correlated with bacterial dysentery incidence. Increased forest coverage can improve the water retention capacity of the soil and reduce soil erosion, thereby protecting the water environment of rivers, lakes, and other water sources, ensuring water quality. This reduces water source pollution, thereby eliminating the possibility of BD diseases being transmitted through waterways (35).

There may be a consensus or consistent trends regarding the positive or negative associations of these related factors. However, the primary associated factors identified can vary slightly depending on the method used. Therefore, this study presents the results of a panel data model, based on four categories of factors, to allow comparison with existing findings. In our study, we found that the impact of meteorological factors may be mitigated by incorporating forest coverage. Consistent with this finding, previous studies have reported that forest coverage can reduce the impact of high temperature levels (36) or rainfall (11) on infectious diseases.

### 4.3 Policy suggestions

To enhance the effectiveness of these measures, especially in the context of intensifying climate change, we recommend that policymakers: (1) prioritize the equitable distribution of healthcare infrastructure, particularly in underserved regions, to ensure broader access to medical services; (2) integrate environmental conservation into public health planning, leveraging the protective role of forest coverage by expanding urban green spaces and restoring natural habitats; and (3) develop targeted economic policies to raise household incomes and improve sanitation standards, thereby reducing population susceptibility to infectious diseases such as BD.

### 4.4 Limitations

A major limitation of this study is its ecological design, which only captures population-level associations rather than individual-level relationships. Therefore, we could not infer causal effects between the studied factors and BD incidence, and the observed correlations may be confounded by unmeasured factors at the individual or community level. Ecological proxy variables cannot capture the uneven distribution within an area or the status of individuals. This measurement error is highly likely to cause attenuation bias in the correlation effect and prevent the model from fully accounting for the complex social environment confounding represented by these proxy variables, thereby posing a risk of residual confounding. Residual confounding can interfere with the judgment of the true causal relationship between the target variable and disease risk and reduce the internal validity of the model. Therefore, the research findings should be interpreted as associations at the macro level rather than precise estimates of effect size.

Additionally, important determinants of BD, such as hygiene practices, household-level drinking water quality, local public health policies, and antimicrobial resistance, were not included due to data constraints. The classic determinants were excluded due to unavailable data, which is highly likely to introduce omitted variable bias. For example, some of the observed correlations in this study (such as GDP per capita) might actually be the confounding manifestations of more direct factors (hygiene practices) that were not controlled for. Future research needs to integrate individual-level survey data to distinguish and verify the underlying micro-level mechanisms driving these macro-level effects. Furthermore, in practical applications, the influence of other factors, such as sanitation facilities, water-borne disease outbreaks, municipal sewage systems, and population mobility, should be considered. This might affect the complete interpretation of the model regarding the drivers of incidence.

The limitations of using a short temporal dimension (5 years) in this panel study include: (1) potential instability of model estimates; (2) estimation bias due to high correlations between years; (3) the use of annual aggregate data, which may mask seasonal variations; (4) limited ability to assess long-term trends or delayed effects; and (5) the “elasticity” or correlation estimated by the model mainly reflects cross-sectional differences between provinces, rather than strong dynamic effects that changed over time. Even if the panel data analysis reveals a correlation between certain variables and the incidence of BD, a causal relationship cannot be established.

The current panel model did not consider spatial autocorrelation. Future research should combine spatial modeling methods to better capture the regional clustering and diffusion mechanisms of diseases. However, differences in monitoring standards and reporting completeness of BD across provinces may affect the cross-regional comparability of the results.

## 5 Conclusion

Using panel data from Chinese provinces between 2014 and 2018, this study quantified the key factors associated with reductions in bacillary dysentery incidence: A 1% increase in the number of beds per 1,000 people in healthcare institutions, GDP per capita, and forest coverage was associated with a 2, 0.8, and 0.6% decrease in BD incidence, respectively. Enhanced regional investment in healthcare resources, economic development, and forest coverage might contribute to reducing the bacillary dysentery burden at the regional level.

## Data Availability

The original contributions presented in the study are included in the article/supplementary material, further inquiries can be directed to the corresponding author.
